# Insecticidal and knockdown resistance status of *Anopheles gambiae* s.l (Diptera: Culicidae) to pyrethroid and organophosphate insecticides in Osun State, Nigeria

**DOI:** 10.1371/journal.pone.0347416

**Published:** 2026-04-20

**Authors:** Lateef Oluwatoyin Busari, Zarat Oyindamola Iwalewa, Olabanji Ahmed Surakat, Adedapo Olufemi Adeogun, Akinlabi Mohammad Rufai, Kamilu Ayo Fasasi, Monsuru Adebayo Adeleke

**Affiliations:** 1 Parasitology and Vector Biology Unit, Department of Animal and Environmental Biology, Osun State University, Osogbo, Osun State, Nigeria; 2 Molecular Entomology and Vector Control Unit, Public Health and Epidemiology Department, Nigerian Institute of Medical Research, Yaba, Lagos State, Nigeria; 3 Pest Management and Toxicology Unit, Department of Animal and Environmental Biology, Osun State University, Osogbo, Osun State, Nigeria; PLOS ONE, UNITED KINGDOM OF GREAT BRITAIN AND NORTHERN IRELAND

## Abstract

Insecticide resistance in malaria vectors remains a global public health problem; however, little is known about resistance levels in Osun State, despite relatively high rates of malaria and distribution of insecticide-treated nets in the area. This study evaluates the resistance status of adult female *Anopheles gambiae* s.l to pyrethroids (permethrin, deltamethrin and alpha-cypermethrin) and an organophosphate (pirimiphos-methyl) insecticides and knockdown resistant (KDR) gene detection in six locations (Ido-Osun, Ipetumodu, Inisa, Ejigbo, Ijebu-Jesha and Ila) across the three senatorial districts in Osun State, Nigeria. Larval sampling was done between 0700hr and 1100hrs weekly between January and December 2022. Collected larvae were reared to the adult stage in the Department of Animal and Environmental Biology laboratory of Osun State University, Osogbo, Nigeria and then identified morphologically using morphological keys. Insecticide bioassay was conducted with permethrin (0.75%), deltamethrin (0.05%), alpha-cypermethrin (0.05%) and pirimiphos-methyl (0.25%) using WHO procedure. The mosquitoes were subjected to molecular analysis to detect the KDR gene. Pirimiphos-methyl showed significantly higher knockdown at 60 minutes (KD60) and achieved 100% mortality compared with the pyrethroids tested (p < 0.05), with no resistance detected across the study areas. Overall, pyrethroid mortality ranged from 40% to 97% across the study sites, indicating suspected to confirmed resistance. The lowest mortality was recorded at Ila for permethrin (86%) and at Ejigbo for alpha-cypermethrin (60%) and deltamethrin (40%)”. In addition, there was the detection of the KDR gene across the study areas. The present study reveals the insecticidal efficacy of pirimiphos-methyl against female *Anopheles gambiae* s.l as compared to pyrethroids. Therefore, there is a need to intensify insecticide resistance surveillance of *Anopheles* in Osun State to plan indoor residual spraying with pirimiphos-methyl and explore the use of PBO or dual active ingredient insecticides treated nets (ITNs) to address the potential impacts of pyrethroid resistance.

## Introduction

Vector-borne diseases pose a great global public health concern. Mosquitoes are regarded as the most dangerous malaria vector [1]. Female *Anopheles* mosquitoes, particularly members of the *Anopheles gambiae* complex, are the primary vectors of human malaria and are of major public health concern due to their highly efficient transmission of malaria and other mosquito-borne pathogens. Malaria accounts for half of the 31.1% of deaths globally [2]. Furthermore, Nigeria recorded 26.8% of almost half of the global cases of malaria in 2022 [2]. Unfortunately, malaria incidence seems to be on the rise in Nigeria [3].

There has been concerted efforts toward combating malaria and other diseases transmitted by *Anopheles spp.* through vector control using insecticide-treated nets (ITNs) and indoor residual spraying (IRS) globally [4]. In addition, the global decline in malaria cases and deaths observed between 2000 and 2015 is hugely attributed to the use of insecticides [5].

The growing resistance of malaria vectors to insecticides poses a significant threat to global malaria control and elimination efforts. Since 2020, intensified surveillance and strategic responses have been implemented worldwide. The WHO Global Malaria Programme has expanded its insecticide resistance database to support evidence-based decision-making and guide vector control strategies under the updated Global Technical Strategy for Malaria 2016–2030 [6]. In sub-Saharan Africa, systematic reviews and meta-analyses have revealed widespread resistance in major vector species, prompting the adoption of integrated vector management and novel insecticide formulations [7]. Meanwhile, regions in Southeast Asia and Latin America have strengthened resistance monitoring and diversified control tools to mitigate the impact of resistance on transmission dynamics [8]. These coordinated efforts indicate the urgency of sustaining innovation and collaboration to preserve the efficacy of malaria interventions.

In Nigeria, insecticide-based interventions are employed as the major mosquito control intervention and reports have shown an increase in resistance to insecticides used for control in various parts of the country with observed resistance of *Anopheles* to the insecticides [9]. Currently, there are nine classes of insecticides employed in *Anopheles* vector control such as pyrethroids, carbamates, organochlorines, organophosphates, neonicotinoids, butenolides, pyrroles and juvenile hormone(JH) hormone mimics [2].

Despite Osun State being a malaria-endemic region in southwestern Nigeria, there remains a significant gap in insecticide resistance data and few publications on resistance levels of *Anopheles gambiae* s.l mosquitoes in this area [10]. In particular, there is limited comparative evidence on the efficacy and potency of different insecticide classes against these vectors. Osun State distributes ITN frequently to residents. This lack of localized resistance profiling poses a challenge to the implementation of effective vector control strategies and threatens the sustainability of current malaria interventions in the region. Therefore, the need for this study to investigate the resistance status of the malaria vector and the comparison of the effectiveness of the current insecticides employed in malaria control programs and interventions, the core of which lies on ITNs and IRS in the state.

## Methodology

### Study area

The study was conducted in a total of six communities across each of three senatorial districts (Osun West, Osun East and Osun Central) of Osun State, Nigeria. Two communities were selected by gridding across each of the stratified senatorial districts: Ido-Osun (N7.783333 and E4.483333) and Ejigbo (N7.90292 and E4.31419) from Osun West; Inisa (N7.84855 and E4.32981) and Ila (N8.019116 and E4.901962) from Osun Central; and Ijebu-Jesha (N7.68267 and E4.81436) and Ipetumodu (N7.521339 and E4.436217) from Osun East.

The state is situated in the tropical rain forest zone of Nigeria with a land area of approximately 14,875sq km with a population of more than 3 million. The state is known majorly for tourism due to the presence of ancient cultural edifices such as the Osun groove, the Osun River and so on, which serve as a cynosure of attraction to tourists globally particularly during the annual Osun festival usually held in August.

Furthermore, the major occupation of the inhabitants of the state is agriculture and trading with few industries as compared to other cities of the country. They are involved in the cultivation of cash crops such as cocoa etc.

The study areas were selected randomly by gridding with a view to making the study holistic by cutting across the three senatorial districts of the state and owing to the endemicity of the state for malaria.

### Ethical clearance

Ethical clearance was obtained from the Department of Health Planning, Research and Statistics, Ministry of Health, Osogbo, Osun State (OSHREC/PRS/569T/174).

### Community entry and mobilization

Prior to commencement of the study, visitation was made to all the study communities to create awareness and enlightenment on the vitality of the study through the community heads and heads of primary health centres. The essence of the study was explained to residents as well as its benefits to the study communities and the state at large.

### Larval sampling

A thorough prospection was conducted to identify the breeding sites of mosquitoes such as gutters, open drains, discarded containers and ground pool in the study communities. The larval sampling of all accessible breeding sites was done weekly between 07:00hr to 11:00hr for 12 months (January 2022 – December 2022). Encountered *Anopheles* mosquito larvae were collected using plastic scoopers and sieves of about 0.55 mm mesh –size into labelled containers and conveyed to the laboratory of the Department of Animal and Environmental Biology, Osun State University, Osogbo, Osun State, Nigeria for further analysis.

### Identification of larva and adult mosquito

All larvae collected were identified morphologically with the aid of a dissecting microscope using the morphological keys described by Hopkins [11]. The larvae were reared and allowed to emerge into adults inside mosquito cages. The emergent adult mosquitoes were identified using the morphological keys described by [12] and Coetzee [13]. After which they were subjected to insecticide bioassay.

### Insecticide susceptibility bioassay

The insecticide susceptibility test was carried out using WHO test tubes in accordance with WHO procedures [2]. Emergent two to three days old unfed female *Anopheles* mosquitoes were exposed to papers impregnated with each of permethrin, deltamethrin, alpha-cypermethrin and pirimiphos-methyl with 0.75%, 0.05%, 0.05% and 0.25% discriminating concentrations (DC) respectively. The bioassays were carried out on 25 female *Anopheles* mosquitoes in 4 replicates and 25 mosquitoes in two replicates for control for 60 minutes. The number of mosquitoes knocked down was recorded every 10 minutes during the 1 hour exposure time. Thereafter, the mosquitoes were transferred to holding tubes and fed with sugar solution. This was done by placing cotton pads soaked in a 10% sugar solution on the surface within the mosquito enclosure as it helps to maintain their health post-bioassay. The final mortality was then recorded after 24 hours and the percentage mortality was determined using susceptibility criteria by the [2]. The number of resistant and susceptible *Anopheles* mosquitoes was recorded.


$$\text{ Percentage mortality}=\frac{\text{Number of treated female mosquitoes dead}}{\text{Total number of treated female mosquitoes }}\text{x 100}$$

The susceptibility status is determined using susceptibility criteria by WHO

### Susceptibility criteria

≥ 98% Mortality – Susceptibility; 90–97% Mortality – Possible resistance; < 90% Mortality Confirmed Resistance [2].

### Detection of KDR (knockdown resistance) gene in *An. gambiae* s.l

#### DNA extraction.

The genomic deoxyribonucleic acid (DNA) was extracted from the whole individual mosquito using genomic DNA purification kit manufactured by the Nigeria Institute of Medical Research (NIMR) BIOTECH. The genomic deoxyribonucleic acid (DNA) from 100 randomly selected mosquitoes was extracted by crushing the head and thorax of individual mosquito placed in 2 ml Eppendorf tube with pestle, then homogenized in 500µl lysis buffer. The mixture was vortexed and incubated at 56℃ for 10 min then centrifuged at 10,000 rpm for 1 minutes, after spinning, 200µl of absolute ethanol was added to the tube. The mixture was transferred into spin column and centrifuged at 10,000 rpm for 30 sec, discard the flow-through and blot the collection tube on a tissue paper. Addition of 500µl of wash buffer 1 to the spin column, then centrifuged at 10,000 rpm for 30 sec follows the discard of flow-through and blots the collection tube on a tissue paper. The spin column was centrifuged again at 12,000 rpm for 3minutes to remove all the traces of ethanol, thereafter; the spin column was placed in another microcentrifuge tube. 50µl of elution buffer was added to the centre of the column then incubate at room temperature for 1–2 mins and centrifuged at 10,000 rpm for 1 minute to elute the DNA. DNA is stored at ˗20℃ for PCR (Polymerase Chain Reaction) amplification.

#### PCR amplification.

Protocol provide by Martinez-Torres D et al 1998 [14] was used during the amplification of extracted DNA for Polymerase Chain Reaction.

The genomic region containing the intron of interest was PCR amplified on 10–50 ng of genomic DNA using primers Dg1 and Dg2. One unit of Taq polymerase and 200 ng of each primer were used in a 100 µl total PCR volume. The prepared pre-mix contained total volume of 15µl; 4 µl of mastermix, 1 µl of d1,d2,d3 and d4, 6 µl of ddH₂O, 1µl of DNA template.

Amplification was performed for 35 cycles with initial denaturation at 94°C for 5 min, denaturation 94°C for 1 min, annealing 54°C for 2 min, extension 72°C for 2 min, final extension 72°C for 10 min.

### Gel electrophoresis

1.5g of agarose gel with 100 ml of Tris-acetate- Ethylene-diamine tetra acetic acid (EDTA) was used in electrophoresed of the PCR product. The agarose was melted in a microwave for about 2 mins and then allowed to cool satisfactorily. The gel was stained with 5µl Ethidium Bromide for the visualization and detection of amplified DNA fragment, after cooling, the gel was poured into a clean well casting chamber and electrophoresis comb had been inserted to create wells into which amplicons were loaded. The cast was placed in the electrophoresis tank containing 1X to cover the gel and wells followed by the removal of the comb from the well. The molecular ladder (5µl) was dispensed into the first well, followed by 7µl of each amplicons, which were appropriately loaded and run at 80v with 150mA for an hour. The gel was viewed and taken under UV transilluminator for documentation.

### Data analysis

The data obtained were analyzed using analysis of variance (ANOVA) to determine the significance in correlation between the % mortality rate in relation to the tested insecticides and across the study areas. The criteria defined by WHO 2022 [2] were used in classifying the mortality rates into resistant and susceptible. A % mortality rate greater than 98% indicates susceptibility, between 97−90% indicates possibility of resistance and less than 90% indicates resistance. A p-value of less than 0.05 (p < 0.05) at a 95% confidence interval (CI) would indicate significance in correlation. All analysis was done using Statistical Package for Social Sciences (SPSS), 2021 version.

## Results

### Knockdown of *An. gambiae* s.l. after 60mins of exposure

**Fig 1:**
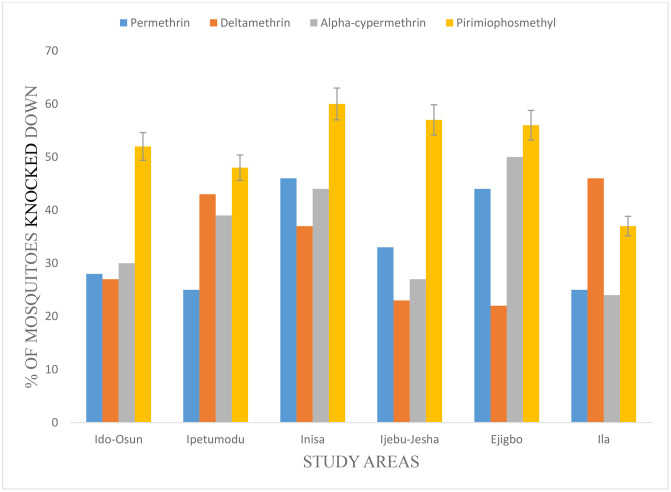
Percentage knockdown after 60mins of exposure to the insecticides. NB Mortality in control groups ranged from 0–4%, confirming assay validity under WHO guidelines.

After 60 minutes of exposure of the adult female *Anopheles* mosquitoes to the insecticide tested, there was an observeable drift in their knockdown efficacy across the study areas. Inisa had the highest knockdown for permethrin and pirimiphos-methyl with 46 (22.9%) and 60 (19.4%) respectively. The lowest knockdown for permethrin, alpha-cypermethrin, deltamethrin, and pirimiphos-methyl was observed in Ipetumodu 25 (12.4%) and Ila 25 (12.4%), Ila 24 (11.2%), Ejigbo 22 (11.1%) and Ila 37 (11.9%) respectively. Furthermore, pirimiphos-methyl, had an overall higher knockdown of 310 adult female mosquitoes than permethrin which had a knockdown of 201 adult female mosquitoes, across the study areas (p < 0.05). The difference in knockdown was significant statistically (Fig 1).

### Post exposure percentage mortality of adult female *An. gambiae* s.l

Post-exposure mortality of adult female *Anopheles gambiae* complex varied across the study locations after 24 hours, indicating differential susceptibility to the insecticides tested. Mortality rates following permethrin exposure ranged from 90–97% in most locations, suggesting possible resistance, except in Ila where a lower rate of 86% indicated confirmed resistance. For deltamethrin, Ejigbo recorded a markedly low mortality rate of 40%, signifying confirmed resistance, while other sites showed rates between 90–97%, consistent with possible resistance. Similarly, alpha-cypermethrin exposure resulted in mortality rates below 90% in Ejigbo (60%) and Ido-Osun (85%), indicating confirmed resistance, whereas other locations exhibited possible resistance. In contrast, pirimiphos-methyl achieved 100% mortality across all sites, demonstrating full susceptibility of the mosquito populations to this insecticide. Overall, the data revealed widespread resistance to pyrethroid insecticides, with pirimiphos-methyl remaining fully effective in all study areas (Table 1–4).

**Table 1:** Post exposure percentage mortality of the adult female *Anopheles gambiae* s.l to the insecticides after 24hrs in the study areas. (a) Permethrin (DC= 0.05%).

Study Community	Environment type	N	Mortality (%)	95% CI	Resistance status
Ido-Osun	Rural	100	97	93.65 - 100.34	R*
Ipetumodu	Rural	100	95	90.72–99.27	R*
Inisa	Rural	100	94	89.34–98.65	R*
Ijebu-Jesha	Peri-Urban	100	96	92.15–99.84	R*
Ejigbo	Peri-Urban	100	96	92.15–99.84	R*
Ila	Peri-Urban	100	86	71.19–92.80	R^C^

Susceptibility criteria: ≥ 98% Mortality = Susceptibility (S); 90–97% Mortality = Possible resistance (R*); < 90% = Confirmed resistance (R^C^) [2]. CI = confidence interval.

Percentages (%) were calculated relative to the 100 adult female mosquitoes exposed in each assay.

**Table 2:** Post exposure percentage mortality of the adult female *Anopheles gambiae* s.l to the insecticides after 24hrs in the study areas. (b) Deltamethrin (DC = 0.05%).

Study area	Environment type	N	Mortality (%)	95% CI	Resistance status
Ido-Osun	Rural	100	90	84.12–95.88	R*
Ipetumodu	Rural	100	96	92.15–99.84	R*
Inisa	Rural	100	96	92.15–99.84	R*
Ijebu-Jesha	Peri-Urban	100	91	85.39–96.60	R*
Ejigbo	Peri-Urban	100	40	30.39–49.60	R^C^
Ila	Peri-Urban	100	95	90.72–99.27	R*

Susceptibility criteria: ≥ 98% Mortality = Susceptibility (S); 90–97% Mortality = Possible resistance (R*); < 90% = Confirmed Resistance (R^C^) [2]. CI = confidence interval.

Percentages (%) were calculated relative to the 100 adult female mosquitoes exposed in each assay.

**Table 3:** Post exposure percentage mortality of the adult female *Anopheles gambiae* s.l to the insecticides after 24hrs in the study areas. (c) Alpha-cypermethrin (DC= 0.05%).

Study area	Environment type	N	Mortality (%)	95% CI	Resistance status
Ido-Osun	Rural	100	85	78.00–91.99	R^C^
Ipetumodu	Rural	100	97	93.65–100.34	R*
Inisa	Rural	100	97	93.65–100.34	R*
Ijebu-Jesha	Peri-Urban	100	87	80.40–93.59	R^C^
Ejigbo	Peri-Urban	100	60	50.39–69.60	R^C^
Ila	Peri-Urban	100	92	86.68–97.31	R*

Susceptibility criteria: ≥ 98% Mortality = Susceptibility (S); 90–97% Mortality = Possible resistance (R*); < 90% = Confirmed Resistance (R^C^) [2]. CI = confidence interval.

Percentages (%) were calculated relative to the 100 adult female mosquitoes exposed in each assay.

**Table 4:** Post exposure percentage mortality of the adult female *Anopheles gambiae* s.l to the insecticides after 24hrs in the study areas. (d) Pirimiphos-methyl (DC= 0.25%).

Study area	Environment type	N	Mortality (%)	95% CI	Resistance status
Ido-Osun	Rural	100	100	100 −100	S
Ipetumodu	Rural	100	100	100–100	S
Inisa	Rural	100	100	100–00	S
Ijebu-Jesha	Peri-Urban	100	100	100–100	S
Ejigbo	Peri-Urban	100	100	100–100	S
Ila	Peri-Urban	100	100	100 − 100	S

Susceptibility criteria: ≥ 98% Mortality = Susceptibility (S); 90–97% Mortality = Possible resistance (R*); < 90% = Confirmed Resistance (R^C^) [2]. CI = confidence interval.

Percentages (%) were calculated relative to the 100 adult female mosquitoes exposed in each assay

### Molecular detection of KDR gene in the female *An. gambiae* s.l. collected in the study areas

**Fig 2:**
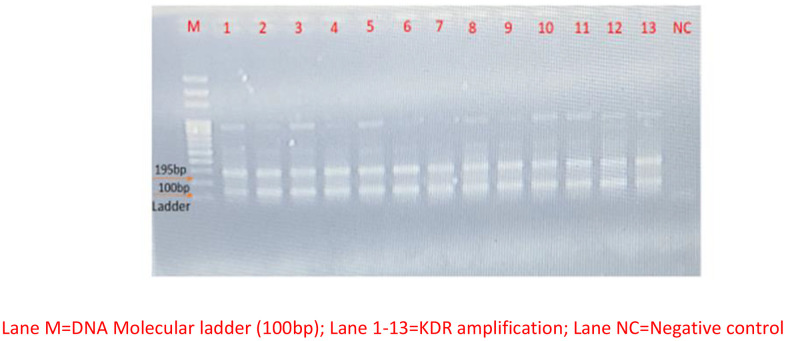
Gel electrophoresis plate showing KDR gene across the study area.

A total of 155 resistant female *Anopheles* mosquitoes to the insecticides used for the insecticide susceptibility bioassay were selected across the study areas. There was a noticeable resistance of the mosquitoes in all the study areas. In addition, the PCR result shows the amplification of the KDR gene at 195 bp in all the 155 resistant female *Anopheles* mosquitoes selected across the study areas as shown in the gel image (Fig 2).

## Discussion

The scourge of insecticide resistance of mosquito vectors, particularly female *Anopheles gambiae* s.l is worrisome due to its public health menace implications in malaria and other *Anopheles*-borne disease transmission.

The use of insecticides has been a major approach in controlling vector-borne diseases. As for malaria, the major insecticides used are the pyrethroids which are employed in ITNs and organophosphates which are used in IRS. The results of the insecticide susceptibility bioassay revealed widespread resistance of *Anopheles gambiae* s.l. to the tested pyrethroid insecticides across all the study locations in Osun State. This observation aligns with recent findings from other regions of Nigeria, where high levels of pyrethroid resistance have been documented in *Anopheles* populations [15]. Similar patterns of emerging resistance have also been reported in other parts of sub-Saharan Africa, including Ghana and Cameroon, highlighting the growing challenge of maintaining the efficacy of pyrethroid-based interventions [16,17].

In addition, this also corroborates earlier reports of permethrin resistance in Osun State and different parts of the country [10,18,19]. Nevertheless, resistance to pyrethroid have been attributed to the wanton misuse of insecticides for agricultural purposes [18,20]. However, environmental factors could have also conferred resistance to the *Anopheles* vectors through climate change and urbanization. Prolonged exposure to pyrethroids, particularly through insecticide-treated nets (ITNs), has been shown to exert strong selection pressure on mosquito populations, leading to resistance via multiple mechanisms. These include target-site mutations such as KDR, metabolic resistance through elevated levels of detoxifying enzymes (e.g., cytochrome P450s), and other genetic adaptations that reduce insecticidal efficacy. Recent studies have shown these mechanisms and highlighted the urgent need for diversified vector control strategies [21,22]. Agricultural practices in the study areas are dominated by cocoa and vegetable farming, which often involves organophosphate and pyrethroid pesticide use, likely contributing to cross-resistance pressure. Furthermore, Adeogun et al., 2019 [23] attributed resistance to paracentric chromosomal inversion 2La. Thus, the need to frequently alter the class of insecticide employed in ITNs with a view to nullifying the resistance mechanism adopted by *An. gambiae* s.l. against insecticides used for mosquito vector control.

However, susceptibility of the mosquitoes was recorded against pirimiphos-methyl in all the study areas (p < 0.05). This conforms to a previous report by [24]. This could possibly be linked to the difference in mode of action of pirimiphos-methyl which is an organophosphate as compared to the other insecticides which belong to the pyrethroid class of insecticides, thus the inability of the mosquitoes to counteract its cidal effect. Likewise, the gross susceptibility recorded could be due to the non-exposure of the vector to pirimiphos-methyl since they are not employed in ITNs which serve as the core method used for vector control.

The detection of KDR gene in all the mosquito samples from the study areas corroborates the emergence of resistance of *An. gambiae* s.l. to the currently employed insecticides used for vector control through IRS and ITNs particularly the pyrethroids. This is consistent with earlier study by Adeogun et al., 2019 [23] who reported resistance in *An. coluzzii* to be attributed to 2La inversion polymorphism. It is a major polymorphic inversion that is associated with adaptive characteristics in *An. gambiae* s.l. [25]. Furthermore, Awolola et al., 2005 [26] had earlier reported the frequency of the KDR allele in *An. gambiae* and not *An. coluzzii* in the southwest. The present study however contradicts this and could be due to mutation as mentioned earlier arising from the indiscriminate use of insecticides for vector control or lack of proper integrated vector management practice arising from long term use of an insecticide etc. The effect of climate change cannot be overruled as it plays a significant role in malaria vector control [27].

### Limitations of the study

The present study has some limitations, such as the lack of reports on the effect of seasonal variation on resistance, characterization of KDR gene frequency, and the lack of *Anopheles gambiae* species-specific data on resistance. However, this study is still useful since it provides some data for Osun State on insecticide resistance patterns, where such information is limited. Follow-up studies would be useful to further understand local resistance dynamics.

## Conclusion

The present study reveals the insecticidal efficacy of the organophosphate (pirimiphos-methyl) against *Anopheles gambiae* s.l despite the resistance of the vectors to pyrethroids in all the study areas. Thus, the need to develop proactive strategies for mosquito insecticide resistance surveillance. This could be through the use of Piperonyl-butoxide (PBO) as a synergist with the pyrethroid insecticide in ITNs. In addition, is the inclusion of pirimiphos-methyl in ITNs and IRS at concentrations that are non-toxic to humans and eco-friendly. Similarly, there is the necessity to frequently interchange the pyrethroid insecticide type employed in ITNs and IRS which undoubtedly is a good integrated vector management control method employable to nullify the resistance potential of the vector as well as their competence. This is paramount to accomplish malaria elimination in the state, the 2030 malaria reduction plan in the country, and the world at large.

## Supporting information

S1 Fig Unedited KDR gel electrophoresis image. (PDF)
