# Supplementary figures and images for: Insecticidal and knockdown resistance status of Anopheles gambiae s.l (Diptera: Culicidae) to pyrethroid and organophosphate insecticides in Osun State, Nigeria
Source: PLoS One. 2026 Apr 20;21(4):e0347416. doi: 10.1371/journal.pone.0347416 (PMC13094965; doi:10.1371/journal.pone.0347416)

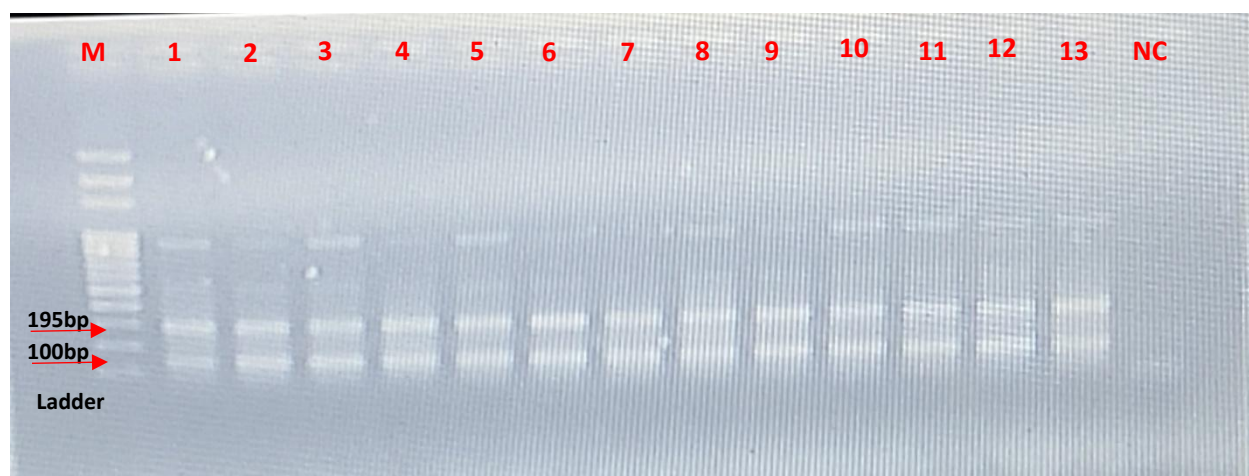

Lane M=DNA Molecular ladder (100bp); Lane 1-13=KDR amplification; Lane NC=Negative control

Supplement: S1 Fig — (PDF) [file pone.0347416.s001.pdf]
